# Transitional Care Interventions in Improving Patient and Caregiver Outcomes After Discharge: A Scoping Review

**DOI:** 10.3390/healthcare13030312

**Published:** 2025-02-04

**Authors:** Giulia Marini, Jessica Longhini, Elisa Ambrosi, Federica Canzan, Hanne Konradsen, Zarina Nahar Kabir

**Affiliations:** 1 Department of Human Sciences, University of Verona, 37129 Verona, Italy; 2 Department of Diagnostics and Public Health, University of Verona, 37134 Verona, Italy; jessica.longhini@univr.it (J.L.); elisa.ambrosi_01@univr.it (E.A.); federica.canzan@univr.it (F.C.); 3 Department of Neurobiology, Care Sciences and Society, Karolinska Institute, 14152 Stockholm, Sweden; hanne.konradsen.01@regionh.dk (H.K.); zarina.kabir@ki.se (Z.N.K.)

**Keywords:** transitional care, continuity of care, caregivers, dyads

## Abstract

**Background.** Caregivers play a fundamental role in the complexity of the transitional process between different healthcare settings. Current research shows that caregiver preparedness can affect the quality and efficacy of post-hospital care, therefore highlighting the need to integrate caregiver roles into the design of transitional care processes. This study aims to map existing evidence on effectiveness of transitional care interventions in improving post-discharge outcomes, as well as the influence of caregiver involvement on both patients and caregivers’ outcomes. **Methods.** Referring to PRISMA-ScR guidelines, a systematic search was conducted between January and February 2024 on Scopus, WoS and PubMed. In order to be included in the systematic search, a study was required to use a RCT design, as well as to describe a transitional care intervention targeting caregivers or patient–caregiver dyads applied in the inpatient setting, lastly the study had to include follow-ups after discharge. There were no limitations on the country or publication year. **Results.** The review has included 51 RCTs of transitional care interventions, discussing caregivers’ roles in improving dyad outcomes after discharge. Although the review highlighted a heterogeneity in the transitional care interventions, it was observed that the interventions shared some common components categorized in the following clusters: need assessments, providing information, psychological support, self-management training, and monitoring or follow-up. **Conclusions.** This review emphasizes the important role of caregivers in the transition from hospital to home, addressing a significant gap in the literature. It highlights the effectiveness of transitional care interventions in improving patients’ quality of life and functional abilities while reducing caregivers’ burden and depression. Further research should focus on assessing the efficacy of these interventions in relation to healthcare utilization, hospital readmission rates, and emergency department visits.

## 1. Introduction

The World Health Organization [1] defines Transitions of Care as “when a patient moves to, or returns from, a particular physical location or makes contact with a health care professional for the purposes of receiving health care”. This includes transitions between community settings, hospitals, and residential care facilities involving various healthcare providers [2].

The transitioning phase between care settings is a vulnerable period for patients and their families [3]. During this time, it is essential to ensure coordination and continuity of care across healthcare contexts and levels, as patients’ needs evolve over time [3,4]. Transitional care encompasses the support provided to patients as they move from one care setting to another, including both pre-hospital preparation and post-discharge follow-up [5,6].

Transitional care interventions aim to assess patients’ and caregivers’ needs, providing tools and support for effective self-management. Coleman et al. [3] suggest that successful transitional care interventions should be grounded in conceptual domains such as discharge assessment, transitional planning, information transfer, patient and family engagement, self-management education, and timely follow-up.

These pillars should be tailored to the specific needs of patients and caregivers, as emphasized by the National Transitions of Care Coalition (NTCC) and Care Transition Interventions (CTI) models, which guide best practices in transitional care.

These frameworks have been implemented across various clinical settings and are supported by literature that documents their core components, such as assessing patient needs during hospitalization and conducting post-discharge follow-ups to actively engage patients and caregivers throughout the transition [7,8,9,10].

The effectiveness of transitional care interventions has been demonstrated in reviews focusing on specific populations, including older adults [6,11,12], patients with stroke [13], heart failure [14], and other chronic conditions. These studies demonstrate that transitional care interventions lead to significant reductions in 30-day readmission rates [14,15], hospital lengths of stay [16], and emergency department visits [14,15], compared to usual care.

Additionally, positive impacts on quality of life, patient satisfaction, and functional status have been reported following these interventions [17,18,19,20], reinforcing the potential of transitional care to improve patient outcomes and healthcare utilization.

Despite substantial research on transitional care, few studies comprehensively examine the complexity of these interventions. This complexity includes the caregiver’s role, which is essential in supporting patients during transitions between healthcare settings [21].

Gitlin and Wolff [22] assert that the caregiver’s role is determined by the patient’s needs, the nature of their relationship with the patient, and their proximity to the patient. Considering that caregiver preparedness significantly affects the quality and efficacy of post-hospital care, it is crucial to comprehend the caregiver’s role in order to design interventions that effectively support them throughout the transitional care process [22]. Involving caregivers in transitional care interventions has been shown to improve patient outcomes, including reduced hospital readmissions and enhanced patient satisfaction and functional status [15,17,20].

Some systematic reviews [15,20] have synthesized caregivers’ roles in transitional care interventions; however, they often focus on specific patient populations and primarily highlight patient outcomes without fully addressing caregiver outcomes.

Recognizing the importance of caregiver involvement, this review aims to address this gap by synthesizing transitional care interventions targeting caregivers or patient–caregiver dyads, with an emphasis on caregiver outcomes.

### Aim

The aim of this study is to synthetize the effectiveness of transitional care interventions in improving post-discharge outcomes, by systematically mapping existing evidence on transitional care interventions involving caregivers and patient–caregiver dyads.

## 2. Methods

A scoping review was performed to synthesize the transitional care interventions aimed at improving patients’ and caregivers’ outcomes after discharge. The scoping review was designed using the PRISMA-ScR (Preferred Reporting Items for Systematic Reviews and Meta-Analyses Extension for Scoping Reviews) guidelines [23] (Supplementary File S1).

### 2.1. Search Strategy

A systematic electronic search was conducted between January and February 2024 for studies published in English and Italian language, on three databases, including Scopus, Web of Science, and PubMed.

The research strategy was developed with the help of a clinical librarian and followed the PRISMA steps: identification, screening, eligibility, and inclusion [23].

The Population (P), Intervention (I), and Outcome (O) (PIO) frameworks guided the development of search terms. Search terms included (P) caregivers, families, and patient–caregiver dyads; (I) all the interventions targeting caregivers or patient–caregiver dyad delivered as transitional care from hospital to home; and (O) all outcomes measured after discharge. The complete research string is attached in a supplementary file (Supplementary File S2).

Furthermore, only articles adopting the following characteristics were included in the research: adopting a randomized control trial design; describing a transitional care intervention targeting caregivers or patient–caregiver dyads applied in the inpatient setting; and finally, including follow-up after discharge. To provide a comprehensive outline of research in the studied field, there were no limitations on country or publication year.

### 2.2. Screening and Selection Process

Studies were entered into the Rayyan software (https://www.rayyan.ai accessed on 28 January 2025) for the screening and selection process. After removing the duplicates, two reviewers independently screened the title and abstract of each study to identify articles meeting the inclusion criteria, a third researcher was consulted in case of discrepancies in eligibility criteria. Subsequently, following the same process, full texts were analyzed in order to decide their inclusion or exclusion in the present study. Lastly, the selection of papers was finalized through a further screening of the reference lists of relevant reviews and studies. Given the inclusion criteria, which focused exclusively on RCT studies to map significant improvements in patients’ and caregivers’ outcomes post-discharge, the gray literature was excluded from this review.

### 2.3. Data Extraction

The data were managed through a database created using a spreadsheet in Microsoft Excel 2018. The data extraction tool was designed to capture the main features of studies meeting the inclusion criteria. The research group defined specific standards to ensure consistency in the reporting of the definitions and variables of interest. The data selected for extraction were Author, Title, Year, Country, Study Design, Aim, Randomization, Arm, Setting, Population, Definition of Caregiver, Inclusion and Exclusion Criteria, Intervention, Time of Follow-up, Primary and Secondary Outcomes, Statistically Significant Results. A single reviewer extracted the data for all included studies using the defined extraction form. All fields of the data extraction form of each article were examined for completeness by a second reviewer.

### 2.4. Data Synthesis

The data were analyzed qualitatively, with narrative analysis, and quantitatively, through absolute and relative frequencies. The analysis of transitional interventions was performed using the classification provided by Hirschman et al. [24]. The transitional care interventions were analyzed to assess the need, information provided, psychological support, self-management training, and monitoring or follow-up. These steps were analyzed in the delivered settings (hospital or home) and the mode of performance, such as face-to-face mode with the booklet support or digital mode with telephone, text message, or video call support.

An additional synthesis has been performed to categorize the outcomes based on delivered intervention. In detail, the outcomes were divided into the interventions delivered to caregivers and to patient–caregiver dyads, and into the interventions provided by multidisciplinary teams, nurses, physiotherapists, psychologists, and other health care professionals.

### 2.5. Search Results

**Figure 1:**
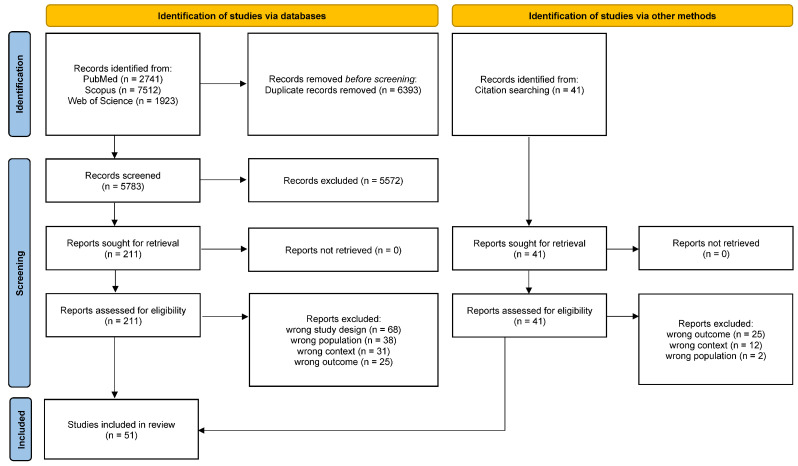
PRISMA flow diagram by Tricco et al. [23].

The search strategy elicited 5783 articles after duplicates were removed. After the initial review of titles and abstracts, 211 studies were deemed eligible for a full-text review. A total of 162 studies were excluded for the following reasons: 68 were not randomized controlled trials (RCTs), 38 did not involve caregivers, 31 did not focus on the inpatient-to-home transition, and 25 did not report primary outcomes in the article (Figure 1).

Following a comprehensive review, a total of 51 randomized controlled trials (RCTs) were deemed eligible for inclusion in the study. These RCTs were considered essential for characterizing the implementation of transitional care interventions aimed at caregivers or patient–caregiver dyads during the transition from hospital to home.

## 3. Results

### 3.1. Characteristics of the Studies

Most of the articles (38/51) were published in the last ten years, between 2014 to 2024. The major country publishing studies on transitional care targeting caregivers or dyads was China, followed by the USA, United Kingdom, and Iran. A multicenter study design was employed in 20 RCTs to assess transitional care interventions implemented during the transition from hospital to home. Of the 51 studies reviewed, 29 involved interventions targeting patient–caregiver dyads, 19 focused exclusively on caregivers, 2 evaluated caregiver interventions conducted in the presence of patients, and 1 study specifically targeted patients in the presence of caregivers. In most studies (22/51), the target population consisted of patients with stroke. The detailed characteristics of each study can be found in Supplementary File S3.

### 3.2. Transitional Care Interventions

All transitional care interventions described in the literature and targeting dyads or caregivers were multi-component ones. The majority of the studies (38/51) described the transitional care intervention as an educational intervention, while fewer included rehabilitation (7/51) or psycho-educational (6/51) components as a part of the transitional intervention. In order to define different components, some studies referred to some conceptual models, such as the Transitional Care Model (TCM) [10] and the Family-Centered Empowerment Model (FCEM) [25].

For the purposes of this review, Hirschman et al.’s [24] classification was used to analyze the components, which included needs assessments (17/51), information provision (27/51), psychological support (12/51), self-management training (46/51), and monitoring or follow-up (24/51). These components were grounded in well-established theories, including Bandura’s “*Self-Efficacy and Individualized Experiential Training*” [9,26,27], Knowles’ “*Adult Learning Theory*” [28], and Cameron and Gignac’s “*Timing It Right Theory*” [29]. A notable feature of these interventions was the significant involvement of caregivers and patient–caregiver dyads in co-designing the interventions, with nearly all studies (49/51) engaging caregivers as integral, active participants. This involvement extended to the development of educational materials [8,30] and shared training experiences [31], as well as contributing to the design of the patient’s plan of care to align with patients’ preferences, values, and goals [29,32,33,34,35,36,37].

These findings underscore the critical importance of continuous caregiver engagement throughout the transitional care process. Reflecting this emphasis, the majority of studies (44/51) integrated both pre- and post-discharge contact, employing a combination of in-person and digital methods (39/51). However, the timing and frequency of interventions demonstrated considerable variability. Of the studies reviewed, five focused exclusively on pre-discharge contact [26,27,32,33,38], one concentrated solely on the post-discharge period [39], and another provided flexibility in intervention timing [40]. The post-discharge period ranged from one week to six months, and the number of intervention contacts varied from a single session [40] to fourteen sessions [41], with the majority of studies (27/51) not specifying the number of contacts.

**Figure 2:**
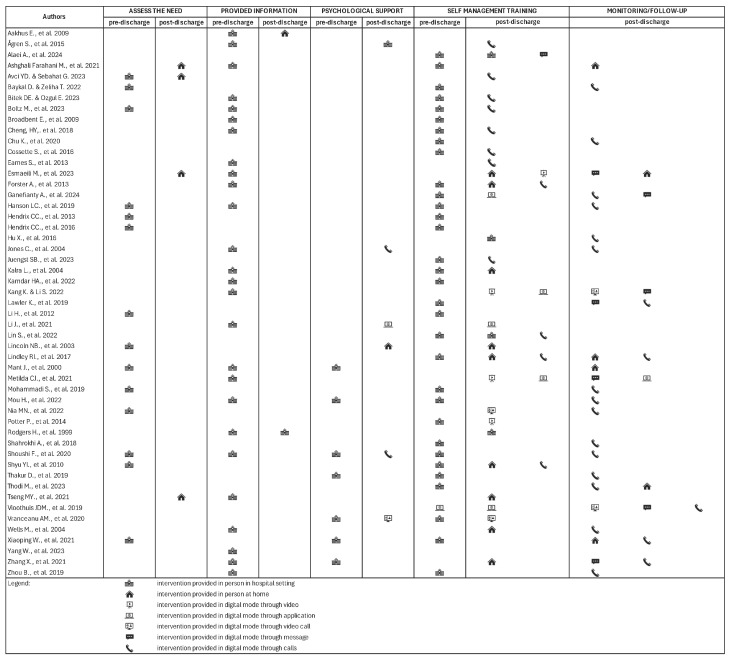
Characteristics of delivered interventions [24].

To gain a deeper understanding of the characteristics of transitional care interventions, the common components are analyzed and presented in detail below, in Figure 2.

#### 3.2.1. Needs Assessment

The needs assessment was focused on caregiver needs, described as either assessing goals or needs and/or assessing caregiver knowledge, skills, and competencies. In all the studies, the results of the needs assessment were used to inform the tailoring of the transitional care intervention. Caregivers’ needs assessments were conducted through consultations and meetings with healthcare professionals to determine the caregiver’s needs for information, knowledge about the home environment, and need for ongoing health services and social services [10,26,27,28,42].

Based on its objective, the needs assessment took place pre- or post-discharge, in the hospital [8,25,26,27,28,33,36,42,43,44,45,46,47] or at home [48,49,50].

The needs assessment was performed pre-discharge in the hospital after consultation with the healthcare professional, and the aim was to assess the information needed about hospitalization or disease information, the treatment process, management emergencies, and symptom prevention. On the other hand, the needs assessment was conducted at home post-discharge to evaluate the home environment and determine the patient’s need for health and social services [49,50].

#### 3.2.2. Providing Information

The information component includes the provision of standard or personalized information about the disease, risk factors, medications, the recovery and rehabilitation process, and the follow-up process.

Twenty-six studies describe providing information in person, during the pre-discharge time, specifically in a meeting with healthcare professionals. Four studies described the information provision process with a standardized or personalized booklet support [44,48,50,51]. The standardized booklet supports the etiology of the disease, the signs, symptoms, and the types of treatments [50], as well as the importance of patient care [48,51]. Only three studies analyze the information provision phase in post-discharge time, at the hospital [52], at home [40], or only with a booklet [53].

#### 3.2.3. Psychological Support

Psychological support was considered an important component to guarantee the quality of a transitional process. The efficacy of psychological support to caregivers was observed when delivered in pre-discharge or post-discharge and in person [8,31,54], through digital support tools [35] or a combination of them [30,30,55].

Jones et al. [30] described psychological support that could be provided in pre-discharge through a booklet that includes exercises on relaxation and coping with stress. Other studies provided pre-discharge psychological support through counseling and reinforcement sessions [54] or interactions focused on difficulties and requests of the families [43]. Xiaoping et al. [8] provided pre-discharge psychological support encouraging caregivers to proactively solve problems through positive communication. On the contrary, Vranceanu et al. [55] focused on educational resilience skills sessions on mindfulness, coping skills, and interactions to cope with the new situation. Shared experiences [31], emotional support [44], and digital tools were used to deliver post-discharge psychological support. For instance, Li et al. [35] provided family and social support through the WeChat platform. Other studies [30,46] offered psychological support through phone calls discussing any problems, while others displayed videos aimed at helping dyads get through the trauma of hospitalization [55].

#### 3.2.4. Self-Management Training

Self-management training was described in 46 studies as a series of educational sessions delivered to caregivers or patient–caregiver dyads to improve the management of the new condition [8], to identify and respond quickly to worsening symptoms [26,27,39,49], to prevent complications [28,35,56] and to understand how to act in emergencies [10,57]. 

Only two studies provided training in group sessions focused on peer support [39] and group discussions [25] involving caregivers [39] and patient–caregiver dyads [25].

Eight studies delivered training using digital tools only, such as phone calls [10,31,58] or video calls to share the educational materials, to discuss their learning progress, and ensure that the contents of the previous sessions were learned [25,55]. In only one case [25], the video calls were delivered to group discussions involving more than one dyad. Other studies provided training through educational videos to do rehabilitation exercises or e-health apps based on self-care management, like dietary management. The only use of digital tools happened in post-discharge time, except for Vloothuis et al. [59], who describe the use of applications in pre- and post-discharge.

#### 3.2.5. Monitoring and Follow-Up

The monitoring of the transitional care process was described in 24 studies. The follow-up phase can be delivered through home visits or through digital tools to monitor and update on the progress and needs, answer questions, and provide support. Only five studies described the follow-up only through home visits [43,48] or a combination of digital and in-person methods [50,60,61].

The majority of studies described follow-up through the use of digital tools (19/51) and phone calls were the most frequent method of follow-up (17/51). Phone calls were conducted by the healthcare professional to caregivers after discharge, to monitor the patient’s progress or to support rehabilitation and discharge compliance. In contrast, Shahrokhi et al. [62] and Vloothuis et al. [59] provided the possibility to call the healthcare professional any time the caregivers desired or when necessary.

However, the timing of contact was diverse and included monthly [36,51], weekly [39,47,50], bi-weekly [30,53], or daily [46,56] contact follow-up; in other cases, the follow-up was carried out only when necessary [59,63].

### 3.3. Roles in Transitional Care Interventions

In facilitating the transitional care interventions, the healthcare professionals were mainly represented by nurses (20/51). In other studies, the interventions were facilitated by a multidisciplinary team (9/51), physiotherapist (2/51), clinical psychologist (3/51), nurse and physician (4/51), researcher (8/51) or other healthcare professionals (5/51), like occupational therapist [58] or ward manager [50]. To deliver the intervention, 10 studies planned to involve expertise and specialized facilitators, defined by years of clinical experience or having a master’s degree as a prerequisite. Ten studies planned specific training for facilitators based on accredited or board-certified training [34,41,45], as well as planned training by a specialized healthcare professional in the organization [29,34,36,42,59,64,65]. These planned training varied from one hour [45] to some days [41].

### 3.4. Outcomes of Transitional Care Interventions

#### 3.4.1. Caregivers’ Outcomes

Almost every study (48/51) included in this research evaluated the caregivers’ outcomes. Caregivers’ depression and caregivers’ burden were the most common outcomes assessed in the studies. The Zarit Burden Interview (ZBI) was the most widely used instrument for assessing carer burden [36,37,39,50,51,57,66], while other instruments including the Caregiver Burden Inventory [48,65,67] developed by Novak and Guest [68] and the Caregiver Burden Scale (CBS) [31,41,60] developed by Elmsthål et al. [69].

Additional psychological outcomes included distress or strain (14/51), burnout and de-personalization (3/51), post-traumatic stress disorder (3/51), psychological well-being (2/51), and feeling of guilt (1/51). 

To assess emotional health, the Geriatric Depression Scale (GDS) [40], General Health Questionnaire (GHQ-30) [40,43,44,52] and Hospital Anxiety and Depression scale (HADS) [36,38,41,58,59] were mainly used.

Functional outcomes such as caregiving ability (9/51), preparedness to deliver home care (5/51), level of competence (4/51), knowledge (3/51), self-efficacy, and coping skills were also examined.

Knowledge and competencies were assessed using tailored instruments like the Knowledge of Stroke Questionnaire [58] and the Fall Prevention Knowledge Survey [70]. Quality of life was commonly measured with instruments such as the Short Form-36 [29,39,42,43,52,66] and EuroQol [61,71] while satisfaction was assessed using the Client Satisfaction Questionnaire [37] or defined Likert scales [44,63,63]. 

**Figure 3:**
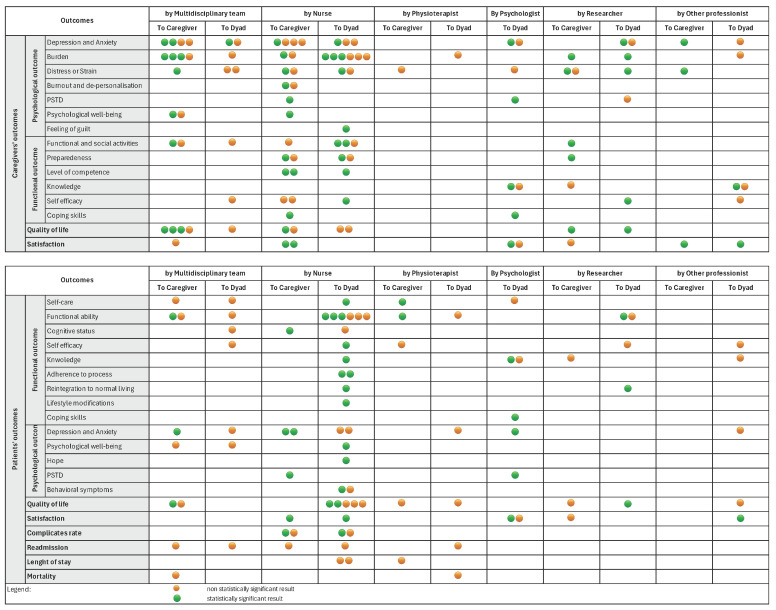
Outcomes evaluated in the studies included.

As outlined in Figure 3, statistically significant results were highlighted for each caregiver outcome based on the intervention delivered. Among the 38 studies evaluating caregiver depression and anxiety, only 7 demonstrated statistically significant improvements [25,38,39,46,55,59,71]. Similarly, 9 out of 23 studies assessing caregiver burden reported statistically significant reductions [9,39,41,48,50,51,57,61,71]. Notably, interventions showing significant outcomes were predominantly caregiver-targeted and delivered by nurses or multidisciplinary teams.

#### 3.4.2. Patients’ Outcomes

Patients’ outcomes were analyzed in 33 studies. Patients’ functional abilities and quality of life were the most common outcomes assessed in the studies. The Modified Rankin Scale [51,59,71] is the most widely used instrument for assessing functional abilities. To assess quality of life, EuroQol five dimensions questionnaire (EQ-5D) [34,41,60,65], EuroQol visual analog scale [71], and Short Form health survey (SF-36) [42,49] were mainly used. Other common results were correlated to functional outcomes and involved self-care (7/51), cognitive status (3/51), self-efficacy (4/51), knowledge (3/51), adherence to process (2/51), reintegration to normal living (2/51), lifestyle modifications (2/51), and coping skills (1/51). Other outcomes were correlated to psychological outcomes and included depression and anxiety, psychological well-being, post-traumatic stress disorder, and behavioral symptoms.

In Figure 3, statistically significant results are highlighted for each patient outcome, differentiated by the delivered intervention. Six out of thirteen studies assessing functional status demonstrated statistically significant results. Similarly, four out of twelve studies assessing patients’ quality of life reported statistically significant improvements (Figure 3). The majority of the studies that reported a significant result included interventions targeted at patient–caregiver dyads and provided by nurses (Figure 3). Other patient outcomes addressed by the studies were: hospital length of stay [33,34], visits to the emergency department or readmissions [10,33,41,57], and mortality [60,71], but these did not show any statistically significant results.

#### 3.4.3. Follow-Up

The follow-up timing varied ranging from 72 h [36] to 12 months after discharge [31,41,42,49,56,63,71]. Although 3 months was the most common follow-up period (24/48), important differences were observed from the initial time of follow-up count: the majority of studies evaluated outcomes after discharge (25/51), while other studies evaluated outcomes after intervention (17/51), after recruitment or randomization (4/51), or after diagnosis (2/51).

## 4. Discussion

This scoping review includes 51 randomized controlled trials (RCTs) of transitional care interventions involving caregivers and patient–caregiver dyads, aimed at improving patient and caregiver outcomes post-discharge. The study underscores the important role that caregivers play in the transition from hospital to home, addressing a significant gap in the literature regarding transitional care interventions involving caregivers.

### 4.1. Transitional Care Interventions and the Caregivers’ Involvement

This scoping review highlights that transitioning interventions from hospital to home share some common components, as categorized by Hirschman et al. [24].

In each component, the involvement of caregivers and patient–caregiver dyads highlights their important role in defining the transitional care process. This process should be grounded in the following similarities: the patient’s and caregiver’s needs, the caregiver’s relationship to the patient, and the caregiver’s proximity to the patient [21,22], in order to define effective transitional interventions.

In light of this, to effectively guide the transitional process, it is fundamental to focus on caregiver involvement by assessing and addressing the caregiver’s needs, through self-management training and personalized information based on those needs.

Therefore, regarding implications for clinical practice, transitional interventions should be planned by a multidisciplinary team and tailored to meet the needs of both the patient and the caregiver, ensuring their active participation during the transition from hospital to home.

However, the ways in which caregivers and patients are involved may differ in the transitional care interventions. The most common contact methods are by phone or in person, either at home or in the hospital setting, and can vary between pre- and post-hospital discharge periods. Although face-to-face contact has traditionally been considered a method for delivering humanized nursing care [72], it is not the primary method used, especially post-discharge. Post-discharge contacts for monitoring and follow-up are often conducted through digital methods such as phone calls, text messages, or video calls.

The present study can be enriched by further research, specifically regarding the possibility of tailoring interventions to match the specific needs of patient–caregiver dyads. Such personalized interventions could include modality (in-person or digital), the use of supportive materials, provision, either individually or in groups, and duration (defining the number and timing of sessions).

Further research should also focus also on determining how caregiver involvement in transitional care interventions can influence both caregivers’ and patients’ outcomes, as well as exploring methods to increase their engagement in the transitional care process.

### 4.2. Outcomes of Transitional Care Interventions

Nurses and multidisciplinary teams are the most commonly involved professionals in the delivery of care transitions [24], contributing to improvements in the psychological and functional outcomes of both caregivers and patients.

The multidisciplinary approach views all team members as collaborators in a care process that prioritizes the needs of patients and their family caregivers [24]. This approach represents an effective strategy for enhancing care transitions and improving both patient and caregiver outcomes [24].

Specifically, depression and caregiver burden are the most frequently evaluated caregiver outcomes, while functional abilities and quality of life are the most relevant patient outcomes.

This finding underscores the importance of selecting outcomes that reflect the impact of transitional care processes on a patient’s functional abilities and a caregiver’s well-being, particularly given the increasing complexity of patient needs and the care required in home settings, as a result of population aging, the prevalence of chronic illnesses, and multiple comorbidities [73].

On the other hand, this finding also highlights the necessity of evaluating and synthesizing the efficacy of interventions in terms of the number of readmissions, visits to the emergency department, and healthcare system costs. This need is emphasized to inform the healthcare system about the effectiveness of transitional care interventions and to promote the increased adoption of structured interventions from hospital to home, engaging caregivers and patient–caregiver dyads.

To enable structured interventions, it is necessary to not only consider the type of outcomes evaluated but also the timing of follow-up assessments, which varied across the studies in terms of the initial time point of follow-up. This heterogeneity represents a limitation of the review. Therefore, future research should focus on defining systematic reviews aimed at evaluating the efficacy of long-term outcomes after a patient’s discharge.

The challenge in evaluating the efficacy of each transitional care component is related not only to the heterogeneity of the studies included, but also to the inherent complexity of the transitional care process. This complexity necessitates careful consideration of other aspects of the intervention, such as the type and number of sessions, the content of the intervention, the strategies employed, the materials provided, the personnel involved, and the setting in which the intervention was delivered.

Furthermore, it would be useful to examine the potential consequences of different components of the interventions, taking into account the complexity of transitional care. This could justify conducting further systematic research, which would address an important gap in the literature by establishing a formal quality appraisal of the studies included.

## 5. Strengths and Limitations

The methodological approach is considered a strength of this review: a broad electronic search of studies was performed, covering all transitional care interventions. The selection, extraction, and analysis of the studies were performed independently by three researchers, with any disagreement resolved by consensus.

However, the choice of inclusion criteria could require some consideration. In detail, exclusively including RCTs could be viewed as both a strength and a limitation of this review. On the one hand, excluding non-RCT studies enabled us to highlight all significant outcomes related to transitional care interventions. On the other hand, a scoping review design would not have necessitated the limitation to RCT study designs, but rather a broader consideration of the gray literature, which would allow for a more comprehensive mapping of evidence related to transitional care interventions.

## 6. Conclusions

This scoping review enabled the identification of key characteristics of transitional care interventions targeting caregivers or patient–caregiver dyads. In this way, the literature gap was addressed by summarizing all transitional care interventions from hospital to home, highlighting the effective outcomes of patient–caregiver dyads.

Despite the heterogeneity of interventions, five common transitional care components were identified in the literature: needs assessments, providing information, psychological support, self-management training, and monitoring or follow-up. In each component, the involvement of caregivers and patient–caregiver dyads emphasized their important role in the transitional care process from hospital to home. In detail, the literature suggests that the involvement of caregivers or patient–caregiver dyads in the transitional care intervention leads to better outcomes for both patients and caregivers.

This review highlights the effectiveness of such interventions on a patient’s quality of life, functional abilities, and the caregiver’s burden and depression.

However, it is necessary to consider the high heterogeneity highlighted in the studies regarding characteristics of interventions, the people involved, the settings where the interventions were provided, and the timing of follow-up interventions.

For this reason, this scoping review could be a precursor to a systematic review needed to address the aspect of heterogeneity highlighted by the studies in the literature and to evaluate the efficacy of healthcare utilization, hospital readmission reduction, and emergency department visits.

## Supplementary Materials

The following supporting information can be downloaded at: https://www.mdpi.com/article/10.3390/healthcare13030312/s1. File S1: PRISMA checklist; File S2: Final query; File S3: Characteristics of studies included.

## References

[1] World Health Organization (2016). Multimorbidity: Technical Series on Safer Primary Care.

[2] Holland D.E., Harris M.R. (2007). Discharge planning, transitional care, coordination of care, and continuity of care: Clarifying concepts and terms from the hospital perspective. Home Health Care Serv. Q..

[3] Coleman E.A., Parry C., Chalmers S., Min S.J. (2006). The Care Transitions Intervention: Results of a Randomized Controlled Trial. Arch. Intern. Med..

[4] Earl T., Katapodis N., Schneiderman S. (2020). Making Healthcare Safer III: A Critical Analysis of Existing and Emerging Patient Safety Practices.

[5] Graham C.L., Ivey S.L., Neuhauser L. (2009). From hospital to home: Assessing the transitional care needs of vulnerable seniors. Gerontologist.

[6] Allen J., Hutchinson A.M., Brown R. (2014). Quality care outcomes following transitional care interventions for older people from hospital to home: A systematic review. BMC Health Serv. Res..

[7] Desai A.D., Popalisky J., Simon T.D. (2015). The effectiveness of family-centered transition processes from hospital settings to home: A review of the literature. Hosp. Pediatr..

[8] Xiaoping W., Yueying G., Jin L., Xiyan T., Qian L., Danping G. (2021). Effects of care transitions intervention mode on the benefit-finding in caregivers for patients with acute cerebral infarction. Signa Vitae.

[9] Lin S., Xiao L.D., Chamberlain D., Ullah S., Wang Y., Shen Y., Chen Z., Wu M. (2022). Nurse-led health coaching program to improve hospital-to-home transitional care for stroke survivors: A randomized controlled trial. Patient Educ. Couns..

[10] Avci D.Y., Gözüm S. (2023). Effects of Transitional Care Model-Based Interventions for Stroke Patients and Caregivers on Caregivers’ Competence and Patient Outcomes: Randomized Controlled Trial. Comput. Inform. Nurs..

[11] Morkisch N., Upegui-Arango L.D., Cardona M.I., Van den Heuvel D., Rimmele M., Sieber C.C., Freiberger E. (2020). Components of the transitional care model to reduce readmission in geriatric patients: A systematic review. BMC Geriatr..

[12] Liebzeit D., Rutkowski R., Arbaje A.I., Fields B., Werner N.E. (2021). A scoping review of interventions for older adults transitioning from hospital to home. J. Am. Geriatr. Soc..

[13] Prvu Bettger J., Alexander K.P., Dolor R.J., Olson D.M., Kendrick A.S., Wing L., Duncan P.W. (2012). Transitional care after hospitalization for acute stroke or myocardial infarction: A systematic review. Ann. Intern. Med..

[14] Vedel I., Khanassov V. (2015). Transitional care for patients with congestive heart failure: A systematic review and meta-analysis. Ann. Fam. Med..

[15] Chartrand J., Shea B., Hutton B., Dingwall O., Kakkar A., Chartrand M., Poulin A., Backman C. (2023). Patient- and family-centred care transition interventions for adults: A systematic review and meta-analysis of RCTs. Int. J. Qual. Health Care.

[16] Saunders S., Killackey T., Kurahashi A., Walsh C., Wentlandt K., Lovrics E., Scott M., Mahtani R., Bernstein M., Howard M. (2019). Palliative care transitions from acute care to community-based care: A systematic review. J. Pain Symptom Manag..

[17] Smith T.O., Pearson M., Pfeiffer K., Crotty M., Lamb S.E. (2019). Caregiver interventions for adults discharged from the hospital: Systematic review and meta-analysis. J. Am. Geriatr. Soc..

[18] Oyesanya T.O., Loflin C., Byom L., Harris G., Daly K., Rink L., Bettger J.P. (2021). Transitions of care interventions to improve quality of life among patients hospitalized with acute conditions: A systematic literature review. Health Qual. Life Outcomes.

[19] Dy S.M., Apostol C., Martinez K.A., Aslakson R.A. (2013). Continuity, coordination, and transitions of care for patients with serious and advanced illness: A systematic review of interventions. J. Palliat. Med..

[20] Van Dijk M., Vreven J., Deschodt M., Verheyden G., Tournoy J., Flamaing J. (2020). Can in-hospital or post-discharge caregiver involvement increase functional performance of older patients? A systematic review. BMC Geriatr..

[21] Ariza-Vega P., Ortiz-Piña M., Kristensen M.T., Castellote-Caballero Y., Jiménez-Moleón J.J. (2019). High perceived caregiver burden for relatives of patients following hip fracture surgery. Disabil. Rehabil..

[22] Gitlin L.N., Wolff J. (2012). Family involvement in care transitions of older adults: What do we know and where do we go from here?. Annu. Rev. Gerontol. Geriatr..

[23] Tricco A.C., Lillie E., Zarin W., O’Brien K.K., Colquhoun H., Levac D., Moher D., Peters M.D.J., Horsley T., Weeks L. (2018). PRISMA Extension for Scoping Reviews (PRISMA-ScR): Checklist and explanation. Ann. Intern. Med..

[24] Hirschman K.B., Shaid E., McCauley K., Pauly M.V., Naylor M.D. (2015). Continuity of care: The transitional care model. Online J. Issues Nurs..

[25] Nia M.N., Mohajer S., Bagheri N., Sarboozi-hoseinabadi T. (2022). The effects of family-centered empowerment model on depression, anxiety, and stress of the family caregivers of patients with COVID-19: A randomized clinical trial. BMC Prim. Care.

[26] Hendrix C.C., Landerman R., Abernethy A.P. (2013). Effects of an individualized caregiver training intervention on self-efficacy of cancer caregivers. West. J. Nurs. Res..

[27] Hendrix C.C., Bailey D.E., Steinhauser K.E., Olsen M.K., Stechuchak K.M., Lowman S.G., Schwartz A.J., Riedel R.F., Keefe F.J., Porter L.S. (2016). Effects of enhanced caregiver training program on cancer caregiver’s self-efficacy, preparedness, and psychological well-being. Support. Care Cancer.

[28] Mohammadi S., Zabolypour S., Ghaffari F., Arazi T. (2019). The effect of family-oriented discharge program on the level of preparedness for caregiving and stress experienced by the family of stroke survivors. Iran. Rehabil. J..

[29] Zhang X., Lin J.L., Gao R., Chen N., Huang G.F., Wang L., Gao H., Zhuo H.Z., Chen L.Q., Chen X.H. (2021). Application of the hospital-family holistic care model in caregivers of patients with permanent enterostomy: A randomized controlled trial. J. Adv. Nurs..

[30] Jones C., Skirrow P., Griffiths R.D., Humphris G., Ingleby S., Eddleston J., Waldmann C., Gager M. (2004). Post-traumatic stress disorder-related symptoms in relatives of patients following intensive care. Intensive Care Med..

[31] Ågren S., Strömberg A., Jaarsma T., Luttik M.L. (2015). Caregiving tasks and caregiver burden; effects of a psycho-educational intervention in partners of patients with post-operative heart failure. Heart Lung.

[32] Broadbent E., Ellis C.J., Thomas J., Gamble G., Petrie K.J. (2009). Can an illness perception intervention reduce illness anxiety in spouses of myocardial infarction patients? A randomized controlled trial. J. Psychosom. Res..

[33] Li H., Powers B.A., Melnyk B.M., McCann R., Koulouglioti C., Anson E., Smith J.A., Xia Y., Glose S., Tu X. (2012). Randomized controlled trial of CARE: An intervention to improve outcomes of hospitalized elders and family caregivers. Res. Nurs. Health.

[34] Zhou B., Zhang J., Zhao Y., Li X., Anderson C.S., Xie B., Wang N., Zhang Y., Tang X., Bettger J.P. (2019). Caregiver-delivered stroke rehabilitation in rural China: The RECOVER randomized controlled trial. Stroke.

[35] Li J., Li Q.P., Yang B.H. (2021). Participatory continuous nursing using the WeChat platform for patients with spinal cord injuries. J. Int. Med. Res..

[36] Boltz M., Mogle J., Kuzmik A., Belue R., Leslie D., Galvin J.E., Resnick B. (2023). Testing an intervention to improve posthospital outcomes in persons living with dementia and their family care partners. Innov. Aging.

[37] Juengst S.B., Wright B., Driver S., Calhoun S., Muir A., Dart G., Goldin Y., Lengenfelder J., Bell K. (2023). Multisite randomized feasibility study of problem-solving training for care partners of adults with traumatic brain injury during inpatient rehabilitation. NeuroRehabilitation.

[38] Kamdar H.A., Gianchandani S., Strohm T., Yadav K., Chou C.Z., Reed L., Norton K., Hinduja A. (2022). Collaborative integration of palliative care in critically ill stroke patients in the neurocritical care unit: A single center pilot study. J. Stroke Cerebrovasc. Dis..

[39] Hu X., Dolansky M.A., Su Y., Hu X., Qu M., Zhou L. (2016). Effect of a multidisciplinary supportive program for family caregivers of patients with heart failure on caregiver burden, quality of life, and depression: A randomized controlled study. Int. J. Nurs. Stud..

[40] Aakhus E., Engedal K., Aspelund T., Selbaek G. (2009). Single session educational program for caregivers of psychogeriatric inpatients: Results from a randomized controlled pilot study. Int. J. Geriatr. Psychiatry.

[41] Forster A., Dickerson J., Young J., Patel A., Kalra L., Nixon J., Smithard D., Knapp M., Holloway I., Anwar S. (2013). A cluster randomized controlled trial and economic evaluation of a structured training program for caregivers of inpatients after stroke: The TRACS trial. Health Technol. Assess..

[42] Shyu Y.I.L., Kuo L.M., Chen M.C., Chen S.T. (2010). A clinical trial of an individualized intervention program for family caregivers of older stroke victims in Taiwan. J. Clin. Nurs..

[43] Mant J., Carter J., Wade D.T., Winner S. (2020). Family support for stroke: A randomised controlled trial. Lancet.

[44] Lincoln N.B., Francis V.M., Lilley S.A., Sharma J.C., Summerfield M. (2003). Evaluation of a stroke family support organizer: A randomized controlled trial. Stroke.

[45] Hanson L.C., Kistler C.E., Lavin K., Gabriel S.L., Ernecoff N.C., Lin F.-C., Sachs G.A., Mitchell S.L. (2019). Triggered palliative care for late-stage dementia: A pilot randomized trial. J. Pain Symptom Manag..

[46] Shoushi F., Janati Y., Mousavinasab N., Kamali M., Shafipour V. (2020). The impact of family support program on depression, anxiety, stress, and satisfaction in the family members of open-heart surgery patients. J. Nurs. Midwifery Sci..

[47] Baykal D., Tülek Z. (2022). The effect of discharge training on quality of life, self-efficacy, and reintegration to normal living in stroke patients and their informal caregivers: A randomized controlled trial. Neurol. Asia.

[48] Ashghali Farahani M., Najafi Ghezeljeh T., Haghani S., Alazmani-Noodeh F. (2021). The effect of a supportive home care program on caregiver burden with stroke patients in Iran: An experimental study. BMC Health Serv. Res..

[49] Tseng M.-Y., Yang C.-T., Liang J., Huang H.-L., Kuo L.-M., Wu C.-C., Cheng H.-S., Chen C.-Y., Hsu Y.-H., Lee P.-C. (2021). A family care model for older persons with hip fracture and cognitive impairment: A randomized controlled trial. Int. J. Nurs. Stud..

[50] Esmaeili M., Dehghan Nayeri N., Bahramnezhad F., Fattah Ghazi S., Asgari P. (2023). Effectiveness of a supportive program on caregiver burden of families caring for patients on invasive mechanical ventilation at home: An experimental study. Creat. Nurs..

[51] Bitek D.E., Erol O. (2023). The effect of discharge training and telephone counseling service on patients’ functional status and caregiver burden after stroke: A randomized controlled trial. Neurol. Asia.

[52] Rodgers H., Atkinson C., Bond S., Suddes M., Dobson R., Curless R. (1999). Randomized controlled trial of a comprehensive stroke education program for patients and caregivers. Stroke.

[53] Cheng H.Y., Chair S.Y., Chau P.C. (2018). Effectiveness of a strength-oriented psychoeducation on caregiving competence, problem-solving abilities, psychosocial outcomes, and physical health among family caregivers of stroke survivors: A randomized controlled trial. Int. J. Nurs. Stud..

[54] Thakur D., Dhandapani M., Ghai S., Mohanty M., Dhandapani S. (2019). Intracranial tumors: A nurse-led intervention for educating and supporting patients and their caregivers. Clin. J. Oncol. Nurs..

[55] Vranceanu A.-M., Bannon S., Mace R., Lester E., Meyers E., Gates M., Popok P., Lin A., Salgueiro D., Tehan T. (2020). Feasibility and efficacy of a resiliency intervention for the prevention of chronic emotional distress among survivor-caregiver dyads admitted to the neuroscience intensive care unit: A randomized clinical trial. JAMA Netw. Open.

[56] Wells M., Harrow A., Donnan P., Davey P., Devereux S., Little G., McKenna E., Wood R., Chen R., Thompson A. (2004). Patient, carer, and health service outcomes of nurse-led early discharge after breast cancer surgery: A randomized controlled trial. Br. J. Cancer.

[57] Ganefianty A., Songwathana P., Damkliang J., Imron A., Latour J.M. (2024). A Mobile Health Transitional Care Intervention Delivered by Nurses Improves Postdischarge Outcomes of Caregivers of Patients with Traumatic Brain Injury: A Randomized Controlled Trial. World Neurosurg..

[58] Eames S., Hoffmann T., Worrall L., Read S., Wong A. (2013). Randomized controlled trial of an education and support package for stroke patients and their carers. BMJ Open.

[59] Vloothuis J.D.M., Mulder M., Nijland R.H.M., Goedhart Q.S., Konijnenbelt M., Mulder H., Hertogh C.M.P.M., van Tulder M., van Wegen E.E.H., Kwakkel G. (2019). Caregiver-mediated exercises with e-health support for early supported discharge after stroke (CARE4STROKE): A randomized controlled trial. PLoS ONE..

[60] Lindley R.I., Anderson C.S., Billot L., Forster A., Hackett M.L., Harvey L.A., Jan S., Li Q., Liu H., Langhorne P. (2017). Family-led rehabilitation after stroke in India (ATTEND): A randomized controlled trial. Lancet.

[61] Thodi M., Bistola V., Lambrinou E., Keramida K., Nikolopoulos P., Parissis J., Farmakis D., Filippatos G. (2023). A randomized trial of a nurse-led educational intervention in patients with heart failure and their caregivers: Impact on caregiver outcomes. Eur. J. Cardiovasc. Nurs..

[62] Shahrokhi A., Azimian J., Amouzegar A., Oveisi S. (2018). The Effect of Telenursing on Referral Rates of Patients with Head Trauma and Their Family’s Satisfaction after Discharge. J. Trauma Nurs..

[63] Kang K., Li S. (2022). A WeChat-based caregiver education program improves satisfaction of stroke patients and caregivers, also alleviates poststroke cognitive impairment and depression: A randomized controlled study. Medicine.

[64] Cossette S., Belaid H., Heppell S., Mailhot T., Guertin M.C. (2016). Feasibility and acceptability of a nursing intervention with family caregiver on self-care among heart failure patients: A randomized pilot trial. Pilot Feasibility Stud..

[65] Chu K., Bu X., Sun Z., Wang Y., Feng W., Xiao L., Jiang F., Tang X. (2020). Feasibility of a nurse-trained, family member-delivered rehabilitation model for disabled stroke patients in rural Chongqing, China. J. Stroke Cerebrovasc. Dis..

[66] Alaei A., Babaei S., Farzi S., Hadian Z. (2024). Effect of a supportive-educational program, based on COPE model, on quality of life and caregiver burden of family caregivers of heart failure patients: A randomized clinical trial study. BMC Nurs..

[67] Mou H., Lam S.K.K., Chien W.T. (2022). Effects of a family-focused dyadic psychoeducational intervention for stroke survivors and their family caregivers: A pilot study. BMC Nurs..

[68] Novak M., Guest C. (1989). Application of a multidimensional caregiver burden inventory. Gerontologist.

[69] Elmståhl S., Malmberg B., Annerstedt L. (1996). Caregiver’s burden of patients 3 years after stroke assessed by a novel caregiver burden scale. Arch. Phys. Med. Rehabil..

[70] Potter P., Pion S., Klinkenberg D., Kuhrik M., Kuhrik N. (2014). An instructional DVD fall-prevention program for patients with cancer and family caregivers. Oncol. Nurs. Forum.

[71] Kalra L., Evans A., Perez I., Melbourn A., Patel A., Knapp M., Donaldson N. (2004). Training carers of stroke patients: Randomized controlled trial. BMJ.

[72] Reyes-Téllez A., González-García A., Martín-Salvador A., Gázquez-López M., Martínez-García E., García-García I. (2024). Humanization of nursing care: A systematic review. Front. Med..

[73] Wagner E.H., Austin B.T., Davis C., Hindmarsh M., Schaefer J., Bonomi A. (2001). Improving chronic illness care: Translating evidence into action. Health Aff..

