# Supplementary material for: Transitional Care Interventions in Improving Patient and Caregiver Outcomes After Discharge: A Scoping Review
Source: Healthcare (Basel). 2025 Feb 4;13(3):312. doi: 10.3390/healthcare13030312 (PMC11817558; doi:10.3390/healthcare13030312)
Supplement: Supplementary file 1 [file healthcare-13-00312-s001.zip › Supplementary File S2. Final query.pdf]

Research question

Are transitional care interventions targeting caregivers, family members, or patient-caregiver dyad effective in improving patients' and caregivers' outcomes after discharge?

Search		Query PubMed	Results 6/Feb/2024
Caregiver	#1	"Caregivers" [Mesh] OR "caregiver*" OR "nonprofessional home care" OR "non-professional home care" OR "informal care" OR "informal caretaker*" OR "informal social support" OR "family" OR "families" OR "dyad*"	1.647.552
Transition of care	#2	"care transition*" OR "transitional care" OR "transition care" OR "transition of care" OR "transitions of care" OR "transitions care" OR "transitioning care" OR "hospital-to-home" OR "hospital to home" OR "posthospital" OR "aftercare" OR ("discharge"[Title/Abstract])	268.759
No neonatal care	#3	(("infants"[Title/Abstract]) OR ("prematur*"[Title/Abstract])) OR ("neonatal"[Title/Abstract])	632.477
	#4	(#1 AND #2) AND NOT #3	16.714
Limit to RCT studies	#5	"trial" OR "trials" OR "experimental" OR "RCT" OR "random*" OR "control group*"	4.835.774
	#6	#4 AND #5	2.850
Limit to language, studies with abstracts	#7	Filters: Abstract, English language	2.741

Final query in PubMed

((("Caregivers" [Mesh] OR "caregiver*" OR "nonprofessional home care" OR "non-professional home care" OR "informal care" OR "informal caretaker*" OR "informal social support" OR "family" OR "families" OR "dyad*") AND ("care transition*" OR "transitional care" OR "transition care" OR "transition of care" OR "transitions of care" OR "transitions care" OR "transitioning care" OR "hospital-to-home" OR "hospital to home" OR "posthospital" OR "aftercare" OR ("discharge"[Title/Abstract]))) NOT ((("infants"[Title/Abstract]) OR ("prematur*"[Title/Abstract])) OR ("neonatal"[Title/Abstract]))) AND ("trial" OR "trials" OR "experimental" OR "RCT" OR "random*" OR "control group*") **Filters**: Abstract, English

Search		Query Scopus	Results 7/Feb/2024
Caregiver	#1	TITLE-ABS-KEY("caregiver*" OR "carer" OR "carers" OR "nonprofessional home care" OR "non-professional home care" OR "informal care" OR "informal caretaker*" OR "informal social support" OR "family" OR "families" OR "dyad*")	2.568.363
Transition of care	#2	TITLE-ABS-KEY("care transition*" OR "transitional care" OR "transition care" OR "transition of care" OR "transitions of care" OR "transitions care" OR "transitioning care" OR "hospital-to-home" OR "hospital to home" OR "posthospital" OR "aftercare" OR "discharge")	927.159
No neonatal care	#3	(TITLE-ABS-KEY (infants) OR TITLE-ABS-KEY (prematur*) OR TITLE-ABS-KEY (neonatal))	1.873.399
	#4	(#1 AND #2) AND NOT #3	22.942
Limit to RCT studies	#5	("trial" OR "trials" OR "experimental" OR "RCT" OR "random*" OR "control group*")	27.963.521
	#6	#4 AND #5	9.709
Filters Article and English language	#7	Filters: Article, English	7.512

Final query in Scopus

(((TITLE-ABS-KEY("caregiver*" OR "carer" OR "carers" OR "nonprofessional home care" OR "non-professional home care" OR "informal care" OR "informal caretaker*" OR "informal social support" OR "family" OR "families" OR "dyad*")) AND (TITLE-ABS-KEY("care transition*" OR "transitional care" OR

"transition care" OR "transition of care" OR "transitions of care" OR "transitions care" OR "transitioning care" OR "hospital-to-home" OR "hospital to home" OR "posthospital" OR "aftercare" OR "discharge"))) AND NOT ((TITLE-ABS-KEY (infants) OR TITLE-ABS-KEY (prematur*) OR TITLE-ABS-KEY (neonatal)))) AND (TITLE-ABS-KEY("trial" OR "trials" OR "experimental" OR "RCT" OR "random*" OR "control group*")) AND (LIMIT-TO (DOCTYPE,"ar")) AND (LIMIT-TO (LANGUAGE,"English"))

**Search**		**Query Web of Science**	**Results 8/Feb/2024**
**Caregiver**	#1	AB=("caregiver*" OR "carer" OR "carers" OR "nonprofessional home care" OR "non-professional home care" OR "informal care" OR "informal caretaker*" OR "informal social support" OR "family" OR "families" OR "dyad*")	1.552.077
**Transition of care**	#2	AB=("care transition*" OR "transitional care" OR "transition care" OR "transition of care" OR "transitions of care" OR "transitions care" OR "transitioning care" OR "hospital-to-home" OR "hospital to home" OR "posthospital" OR "aftercare" OR "discharge")	475.301
**No neonatal care**	#3	AB=("infant" OR "neonatal" OR "premature")	395.893
	#4	(#1 AND #2) AND NOT #3	10.944
**Limit to RCT studies**	#5	ALL=("trial" OR "trials" OR "experimental" OR "RCT" OR "random*" OR "control group*")	8.361.133
	#6	#4 AND #5	2.217
**Filters Article and English language**	#7	Filters: Article, English	1.923
