# Supplementary material for: Transitional Care Interventions in Improving Patient and Caregiver Outcomes After Discharge: A Scoping Review
Source: Healthcare (Basel). 2025 Feb 4;13(3):312. doi: 10.3390/healthcare13030312 (PMC11817558; doi:10.3390/healthcare13030312)
Supplement: Supplementary file 1 [file healthcare-13-00312-s001.zip › Supplementary File S3. Characteristics of studies included.pdf]

Author (year)	Rationale	Type of intervention	Strategies used	Method to delivery (pre-discharge)	Method to delivery (after discharge)	Materials	Delivered to	Target to	By whom (expertise)	Outcomes	Follow-up (months)
Aakhus et al. (2009)	ND	education	Frontal lecture	In person		None	Caregivers	Psychogeratric	Multidisciplinary team (ND)	**Caregiver**= psychological well-being; depression<br>**Patient**=none	3 after discharge
Ågren et al. (2015)	ND	psycho-education	Sharing experience + Discussion	In person	In person + digital (phone)	None	Dyads	Heart failure	Multidisciplinary team (ND)	**Caregiver**= burden; tasks performed; emotional support<br>**Patient**=none	3-12 after discharge
Alaei et al. (2024)	COPE model	education	Brainstorming + Encouraging	In person	In person + digital (whatsapp)	Booklet	Caregivers	Heart failure	Researcher (ND)	**Caregiver**= quality of life; burden<br>**Patient**= none	3 after discharge
Ashghali Farahani et al. (2021)	ND	education	Q/A	In person	In person	Booklet	Caregivers	Stroke	Researcher (experienced)	**Caregiver**= burden;<br>**Patient**= none	2 weeks after discharge
Avci & Sebahat (2023)	Transitional Care model	education (personalised)	Q/A	In person	In person + digital (phone, website)	None	Caregivers	Stroke	Nurse (ND)	**Caregiver**= competence; preparedness; burnout<br>**Patient**= readmission; frequency of pressure ulcers	3 after discharge
Baykal & Zeliha (2022)	ND	education (personalised)	ND	In person	Digital (phone)	Webpage or booklet	Dyads	Stroke	Researcher (ND)	**Caregiver**= changes in quality of life (physical and mental component); self-efficacy<br>**Patient**= changes in quality of life (physical and mental component); self-efficacy; reintegration to normal living	3 after discharge
Bitek & Ozgul (2023)	ND	education	ND	In person	Digital (phone)	Booklet	Dyads	Stroke	Researcher (ND)	**Caregiver**= burden<br>**Patient**= ADL; Functional status	3 after discharge
Boltz et al. (2023)	Social Cognitive Theory	education	Coaching	In person	Digital (phone)	None	Dyads	Dementia	Nurses (standard training)	**Caregiver**= preparedness; anxiety; strain; burden<br>**Patient**= return to baseline; physical function; delirium; depression	72 hours, 6 after discharge
Broadbent et al. (2009)	Common-Sense model of illness	education	ND	In person	\	None	Dyads	Heart failure	Clinical psychologist (ND)	**Caregiver**= ilness perception; expectations for recovery; anxiety; worry<br>**Patient**= none	1 week after discharge
Cheng et al. (2018)	Caregiving Stress Process model	education	ND	In person	Digital (phone)	Booklet	Caregivers	Stroke	Nurse (experienced)	**Caregiver**= competence; problem-solving coping abilities; depression; burden; perceived social support; family functioning; perceived physical health; incidence of caregiving- related injuries<br>**Patient**= none	1-3 after intervention
Chu et al. (2020)	ND	education + rehabilitation	ND	In person	Digital (phone)	Booklet	Dyads	Stroke	Nurses (standard training)	**Caregiver**= burden<br>**Patient**= functional status; quality of life	3-6 after discharge
Cossette et al. (2016)	Self-determination Theory	education (personalised)	Role playing + Role modelling	In person	Digital (phone)	Check-list	Dyads	Heart failure	Nurses (standard training)	**Dyad**= General self-care management; Self-care; Perceived competence to manage heart failure; motivation; level of support to the patient perceived by caregivers; caregiver's perception of the support they provided to the patient	1 after discharge
Eames et al. (2013)	Health Belief model	education (personalised)	Discussion + Verbal reinforcement	In person	Digital (phone)	Booklet (personalised)	Dyads	Stroke	Occupational therapist (experienced)	**Dyad**= knowledge; self-efficacy; anxiety; depression; ratings of importance of information; feelings of being informed; satisfaction with information<br>**Caregiver**= burden<br>**Patient**= quality of life	3 after discharge
Esmaeili et al. (2023)	ND	education (personalised)	Feedback + Discussion + Q/A	In person	In person + digital (Whatsapp)	Booklet + educational video	Dyads	Invasive mechanical ventilation	Ward manager (ND)	**Caregiver**= burden	3 after discharge
Forster et al. (2013)	London Stroke Carer Training Course	education (personalised)	Demostration+ Frontal lecture	In person	In person or digital (phone)	None	Caregivers	Stroke	Multidisciplinary team (standard training)	**Caregiver**= burden; self-report measures of social restriction; psychological well-being; health state; death; hospitalization and institutionalization	6-12 after discharge

Author (year)	Rationale	Type of intervention	Strategies used	Method to delivery (pre-discharge)	Method to delivery (after discharge)	Materials	Delivered to	Target to	By whom (expertise)	Outcomes	Follow-up (months)
										**Patient**= functional independence; psychological well-being; health state; ADL; functional ability and health-related quality of life; death; hospital readmission and institutionalization	
Ganefianty et al. (2024)	ND	education	Flip-charts+ Demostrations	In person	Digital (phone, online chat)	m-health application	Caregivers	Head trauma	Physician + nurses (experienced)	**Caregiver**= stress transition; burden **Patient**= readmission	2 weeks-1 after discharge
Hanson et al. (2019)	ND	consultation	ND	In person	Digital (phone)	Booklet	Dyads	Dementia	Physician + nurses (standard training)	**Caregiver**= distress **Patient**= hospital or emergency department visits 60days; comfort	1-2 after discharge
Hendrix et al. (2013)	Self-Efficacy and Individualized Experiential Training model	education (personalised)	Interactive training + Demostration + Feedback	In person	/	Booklet	Caregivers (patient presence)	Cancer	Nurse (ND)	**Caregiver**= self-efficacy; depression, anxiety, quality of life **Patient**= functional status; comorbidities; symptoms	2 weeks-1 after discharge
Hendrix et al. (2016)	Self-Efficacy and Individualized Experiential Training model	education (personalised)	Interactive training + Demostration + Feedback	In person	/	Booklet	Caregivers (patient presence)	Cancer	Nurse (ND)	**Caregiver**= self-efficacy (managing symptoms and stress); preparedness; anxiety; depression; burden **Patient**= 30-day rates of ED visits; functional status and well being	2 weeks-1 after discharge
Hu et al. (2016)	ND	education	Q/A + Group discussion	/	In person + digital (phone)	Booklet	Caregivers	Heart failure	Multidisciplinary team (ND)	**Caregiver**= burden; quality of life (physical and mental); depression	3-6 after discharge
Jones et al. (2004)	ND	rehabilitation+ psychological	ND	In person	Digital (phone)	Booklet	Dyads	Post critically	Researcher (ND)	**Caregiver**= anxiety; depression; Post Traumatic Stress Disorder (PTSD)-related symptom	2-6 after discharge
Juengst et al. (2023)	Obesity-Related Behavioral Intervention model	education	ND	In person	Digital (phone)	Booklet	Caregivers	Head trauma	Multidisciplinary team (master level education)	**Caregiver**= satisfaction; engagement; depressive symptoms; burden; positive aspects of caregiving	1-6 after discharge
Kalra et al. (2004)	ND	education	ND	In person	In person	None	Caregivers	Stroke	Multidisciplinary team (ND)	**Caregiver**= burden; function and social activities; anxiety; depression; quality of life **Patient**= mortality; institutionalization; disability; personal self-care; function and social activities; anxiety; depression; quality of life	3-12 after stroke onset
Kamdar et al. (2022)	ND	education	ND	In person	/	None	Caregivers	Stroke	Other healthcare professional (ND)	**Dyad**= anxiety; depression; Post Traumatic Stress Disorder (PTSD)	3 after discharge
Kang & Li (2022)	ND	education	ND	In person	Digital (We-chat)	We-Chat platform	Caregivers	Stroke	Nurses (standard training)	**Caregiver**= satisfaction **Patient**= cognitive impairment; anxiety; depression; satisfaction	3-6-9-12 after intervention
Lawler et al. (2019)	ND	education	ND	In person	Digital (phone or e-mail)	None	Caregivers	Erderly	Physiotherapists (experienced)	**Caregiver**= strain **Patient**= falls-related self-efficacy; physical activity; quality of life; capability-related quality of life; ADL; discharge destination; length of stay	5 weeks after intervention
Li et al. (2012)	COPE model	education	ND	In person	/	Audio-taped + written materials	Dyads	Erderly	Nurse (ND)	**Caregiver**= emotional coping anxiety; emotional coping worry; emotional coping, depression; functional coping, quality/type of care; preparedness for follow-up care; role rewards; role strain; quality of the relationship between caregivers and patients; beliefs coping process **Patient**= rate and duration of dysfunctional syndromes, length of hospital stay, readmission 15days; functional status; depression; cognitive status	2 weeks, 2 after discharge

Author (year)	Rationale	Type of intervention	Strategies used	Method to delivery (pre-discharge)	Method to delivery (after discharge)	Materials	Delivered to	Target to	By whom (expertise)	Outcomes	Follow-up (months)
Li et al. (2021)	ND	education	ND	In person	Digital (We-chat)	We-Chat platform	Dyads	Neuro critically	Nurse (ND)	**Caregiver**= family function; health behavior **Patient**=complications rate (pressure sores, lung, urinary system, joint contracture, muscle atrophy, deep vein thrombosis)	3-6 after discharge
Lin et al. (2022)	Self-Efficacy Individualized Experiential Training model	education	ND	In person	Digital (phone)	None	Dyads	Stroke	Nurse (ND)	**Caregiver**= burden **Patient**= self-efficacy; quality of life; stroke-related knowledge	3-6 after interventions
Lincoln et al. (2003)	ND	education	ND	In person	In person	Booklet	Dyads	Stroke	Clinical psychologist (ND)	**Caregiver**= mood; strain; activities of daily living; perceived knowledge; perceived satisfaction **Patient**= mood; personal self-care; activities of daily living; perceived knowledge; perceived satisfaction	4-9 after recruitment
Lindley et al. (2017)	ND	education	ND	In person	Digital (phone)	None	Dyads	Stroke	Physiotherapists (ND)	**Caregiver**= anxiety and depression; burden **Patient**= dead or dependent; functional dependency; recovery; place of residence; quality of life; anxiety and depression	3-6 after randomisation
Mant et al. (2000)	ND	psychological	Interaction of experiences	In person	In person	None	Caregivers	Stroke	Researcher (ND)	**Caregiver**= knowledge about stroke; social activities; quality of life; satisfaction with services; emotional health; strain **Patient**= knowledge about stroke; social activities; quality of life; satisfaction with services; anxiety; depression; disability; handicap	6 after stroke
Metilda et al. (2021)	ND	education + monitoring	ND	In person	Digital (app)	Aimeo app	Dyads	Post surgery	Nurse or physician (ND)	**Caregiver**= medication compliance; level of confidence in lifestyle modifications **Patient**= medication compliance; level of confidence in lifestyle modifications; revisits due to complications; level of satisfaction	1-2 after discharge
Mohammadi et al. (2019)	Adult Learning Theory	education	ND	In person	Digital (phone)	None	Caregivers	Stroke	Researcher (ND)	**Caregiver**= stress experienced; preparedness	2 after intervention
Mou et al. (2022)	Double ABC-X model	education	ND	In person	Digital (phone)	None	Dyads	Stroke	Nurses (experienced)	**Caregiver**= burden; competence; coping; emotional health; family functioning; dyadic relationship **Patient**= survivors' functioning	1 after intervention
Nia et al. (2022)	Family-Centered Empowerment Model	education	ND	In person	Digital (online meeting)	Whatsapp	Dyads	COVID-19	Researcher (ND)	**Caregiver**= depression; anxiety; stress	5 days after intervention
Potter et al. (2014)	Transactional Model	education	ND	In person	Digital (DVD)	DVD	Dyads	Cancer	ND	**Caregiver**= awareness; perception of general risk factors for falling; Knowledge of the fall-prevention **Patient**= awareness; perception of general risk factors for falling; Knowledge of the fall-prevention	1 week, 1-3 after discharge
Rodgers et al. (1999)	ND	education	ND	In person	In person	None	Dyads	Stroke	Multidisciplinary team (ND)	**Caregiver**= perceived health status; knowledge of stroke; emotional health **Patient**= knowledge of stroke; depression; anxiety; disability; handicap	6 after stroke
Shahrokhi et al. (2018)	ND	education	ND	In person	Digital (phone)	Booklet	Caregivers	Head Trauma	Nurse (ND)	**Caregiver**= satisfaction **Patient**= referrals rate	3 after discharge
Shoushi et al. (2020)	ND	education (personalised)	Indiviual training	In person	Digital (phone)	Booklet	Caregivers	Post surgery	ND	**Caregiver**= depression, anxiety, stress; satisfaction	2 weeks after discharge
Shyu et al. (2010)	ND	education	ND	In person	Digital (phone) + In person	None	Dyads	Stroke	Nurses (standard training)	**Caregiver**= quality of care; quality of life (bodily pain, general health perceptions, vitality, social functioning, role limitation for emotional	1-3-6-12 after discharge

Author (year)	Rationale	Type of intervention	Strategies used	Method to delivery (pre-discharge)	Method to delivery (after discharge)	Materials	Delivered to	Target to	By whom (expertise)	Outcomes	Follow-up (months)
										problem, general mental health, physical functioning) **Patient**= self-care ability; quality of life (bodily pain, general health perceptions, vitality, social functioning, role limitation for emotional problem, general mental health, physical functioning)	
Thakur et al. (2019)	ND	education	Counselor + Reinforcement	In person	Digital (phone)	None	Dyads	Cancer	Nurse (ND)	**Caregiver**= severity of distress **Patient**= number and severity of behavioral symptoms	1 after discharge
Thodi et al. (2023)	ND	education	Reinforcement + Discussion	In person	Digital (phone) + in person	None	Dyads	Heart failure	Nurses (standard training)	**Caregiver**= burden; feeling of guilt; quality of life (about mobility, daily activities, pain/discomfort, anxiety/depression)	6 after discharge
Tseng et al. (2021)	Interdisciplinary Care model	education (personalised)	Suggestion + Discussion	In person	In person	None	Dyads	Erderly	Physician + nurses (standard training)	**Caregiver**= competence; self-efficacy **Patient**= self-care ability; nutritional status assessment; self-rated health	1-3-6-12 after discharge
Vloothuis et al. (2019)	TIDieR guidelines	rehabilitation	Training + Encouraging	In person	Digital (phone, online meeting, email) + In person	E-health app	Dyads	Stroke	Multidisciplinary team (standard training)	**Caregiver**= strain; quality of life; anxiety; depression; fatigue; self-efficacy **Patient**= mobility; depression; anxiety; self-efficacy; fatigue; motor impairment; psychosocial functioning; walking distance; comfortable walking speed; functional mobility; static and dynamic abilities; performance in activities of daily living; activities actually performed; degree of disability or dependence in the daily activities	2-3 after randomisation
Vranceanu et al. (2020)	Non specified theoretical framework	education	Training	In person	Digital (live video)	None	Dyads	Neuro critically	Clinical psychologist (ND)	**Caregiver=** depression and anxiety; PTSD; mindfulness; coping skills **Patient**= depression and anxiety; PTSD; mindfulness; coping skills **Dyad**= interpersonal interactions	3 after discharge
Wells et al. (2004)	ND	education	Training	In person	Digital (phone) + In person	None	Patients (caregivers presence)	Cancer	Nurse (ND)	**Caregiver**= burden **Patient=** quality of life	2 weeks, 1-12 after discharge
Xiaoping et al. (2021)	Care Transitions Intervention model	education + psychological	Training	In person	In person + digital (phone)	None	Caregivers	Stroke	Physician + nurses (experienced)	**Caregiver**= sense of benefit-finding; quality of life	1 after discharge
Yang et al. (2023)	ND	education	Training	In person	Digital (online audiotaped)	None	Dyads	Heart failure	Nurse (ND)	**Caregiver**= anxiety; depression; family function **Patient**= hope; well-being; quality of life; family function	1 weeks, 1-2 after discharge
Zhang et al. (2021)	Hospital-Family Holistic Care model	education + psychological	Q/A	In person	Digital (phone + We-Chat)	Booklet + educational video	Caregivers	Cancer	Multidisciplinary team (standard training)	**Caregiver**= care ability; psychological distress; quality of life	3-6 after discharge
Zhou et al. (2019)	Motor Relearning Theory	education + rehabilitation	Training + Motivation	In person	Digital (phone)	Educational video	Dyads	Stroke	Nurses (standard training)	**Caregiver**= burden **Patient**= physical functioning; mobility; health-related quality of life; length of hospital stay	3-6 post randomisations

Legend = Not defined (ND); Question-Answer (Q/A); Creativity, Optimism, Planning and Expert advice model (COPE model); Post Traumatic Stress Disorder (PTSD)
